# Human subcutaneous dirofilariasis: the ‘migrating’ skin tumor

**DOI:** 10.1080/23320885.2021.2002154

**Published:** 2021-11-14

**Authors:** Michelle Alexandra Mistry, Jens Hoejvig, Marie Helleberg, Christen Rune Stensvold, Pikka Jokelainen, Anders Noehr, Christian Bonde

**Affiliations:** a Department of Plastic Surgery, Breast Surgery and Burns Treatment, Copenhagen University Teaching Hospital, Rigshospitalet, Denmark; b Department of Infectious Diseases, Copenhagen University Teaching Hospital, Copenhagen, Denmark; c Department of Bacteria, Parasites and Fungi, Infectious Disease Preparedness, Statens Serum Institute, Copenhagen, Denmark; d Department of Otorhinolaryngology, Head and Neck Surgery & Audiology, Copenhagen University Teaching Hospital, Copenhagen, Denmark

**Keywords:** *Dirofilaria*, dirofilariasis, Northern Europe, Vector-borne diseases, subcutaneous nodules

## Abstract

A 46-year-old woman presented with facial pain and discomfort. Diagnosis of subcutaneous dirofilariasis was reached after several months from symptom onset. Dirofilariasis should be suspected, also in non-endemic areas, in patients with a migrating subcutaneous nodule. Plastic surgery is preferred, as the face is often involved.

## Introduction

*Dirofilaria* spp. are vector-borne parasitic nematodes, some of which are zoonotic [1]. From the Nordic countries, a small number of apparently imported human dirofilariasis cases [2,3] and one autochthonous human dirofilariasis case [4] have been reported. In Europe, especially *D. repens* has been spreading towards the north [5–8]. Dogs serve as the main reservoir of *D. repens* and can remain microfilaremic for months to years [2,9]. In the mosquitoes, microfilariae mature into infective larval stages, which can be transmitted to a new host when a mosquito takes a blood meal. In humans, dirofilariasis caused by *D. repens* can cause intermittent, painful erythema and itching as well as migrating subcutaneous lesions [3].

Higher awareness of *Dirofilaria* infections is required in the medical field [10]. Surgeons may initially mistake dirofilariasis for malignant disease if they are unacquainted with the infection. This can lead to significant distress for the patient and initial mismanagement and may include the unnecessary use of antibiotics [11,12]. Knowledge on (i) the typical location/migration of the nematode, (ii) whether *Dirofilaria* spp. are endemic to the region, (iii) the travel history of the patient, as well as (iv) the clinical symptoms associated with infection are all essential to establishing a diagnosis of dirofilariasis. *Dirofilaria repens* is often noted in the face, particularly around the eyes. High-resolution ultrasound imaging is useful for detecting movements of the parasite in subcutaneous nodules [13]. Distinguishing *Dirofilaria* from other nematodes mainly relies on morphology (light microscopy and scanning electron microscopy) and/or DNA-based analyses [9,14]. Subcutaneous dirofilariasis should be treated by extraction of the nematode or surgical removal of the nodule. The benefits of anthelminthic drugs such as ivermectin are not clear; however, such drugs might help to stop the migration of the parasite [14,15].

## Case presentation

**Figure 1.**
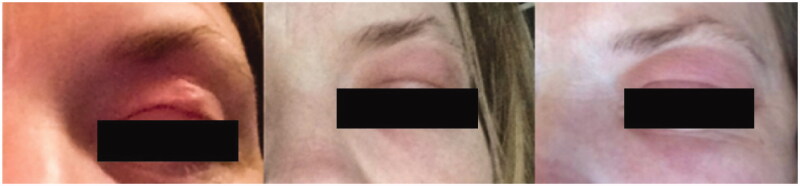
Progressive erythema of the left periorbital area. 1, 2, and 3 March 2018.

**Figure 2.**
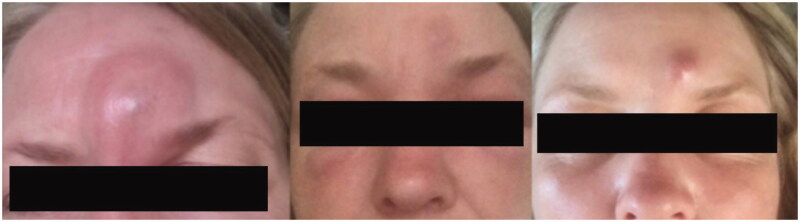
Progression after initiation of oral penicillin. Initially the forehead swelled, accompanied by generalized oedema of the face and the appearance of a red protrusion in the same area.

**Figure 3.**
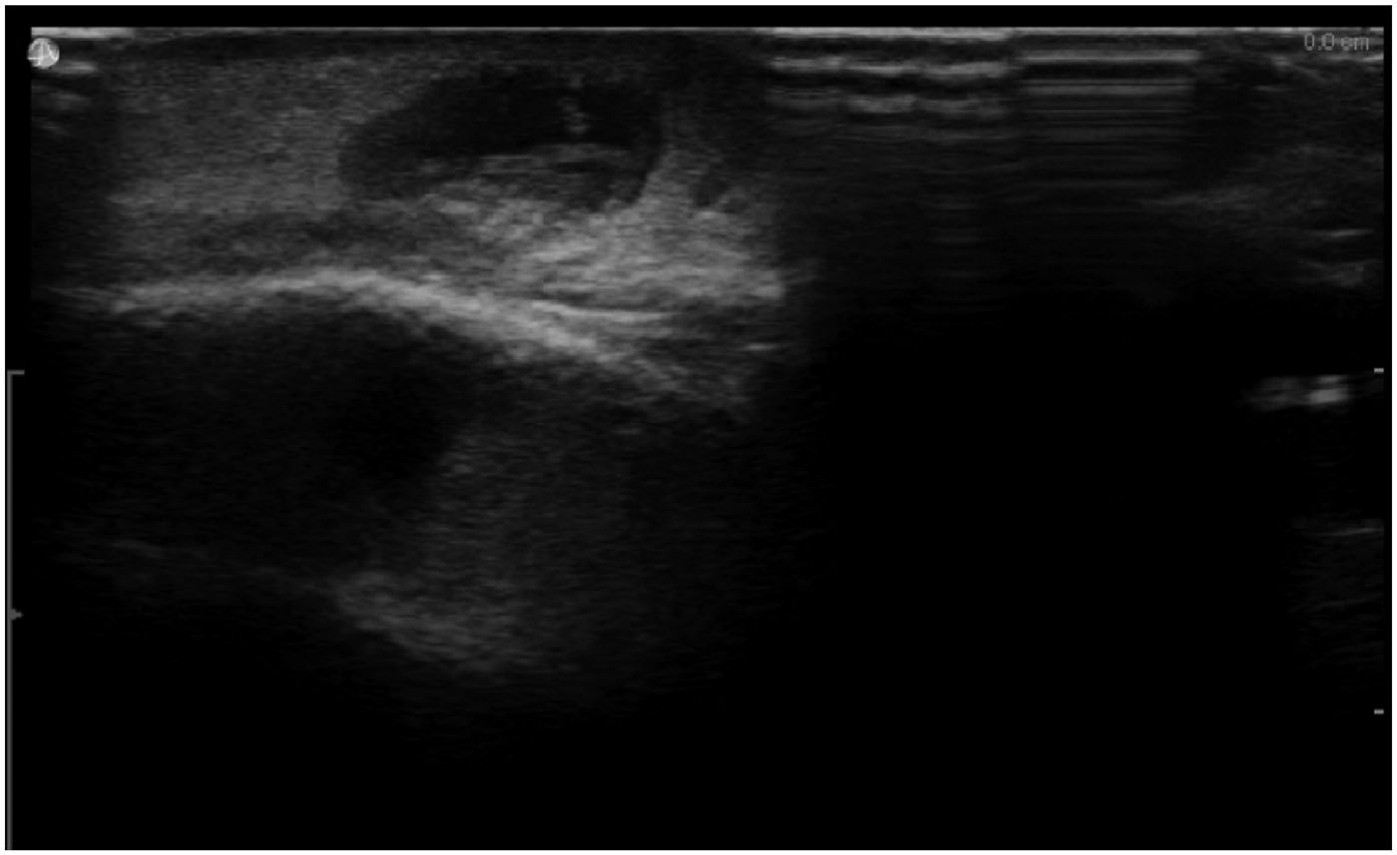
An ultrasound image of the motile nematode.

**Figure 4.**
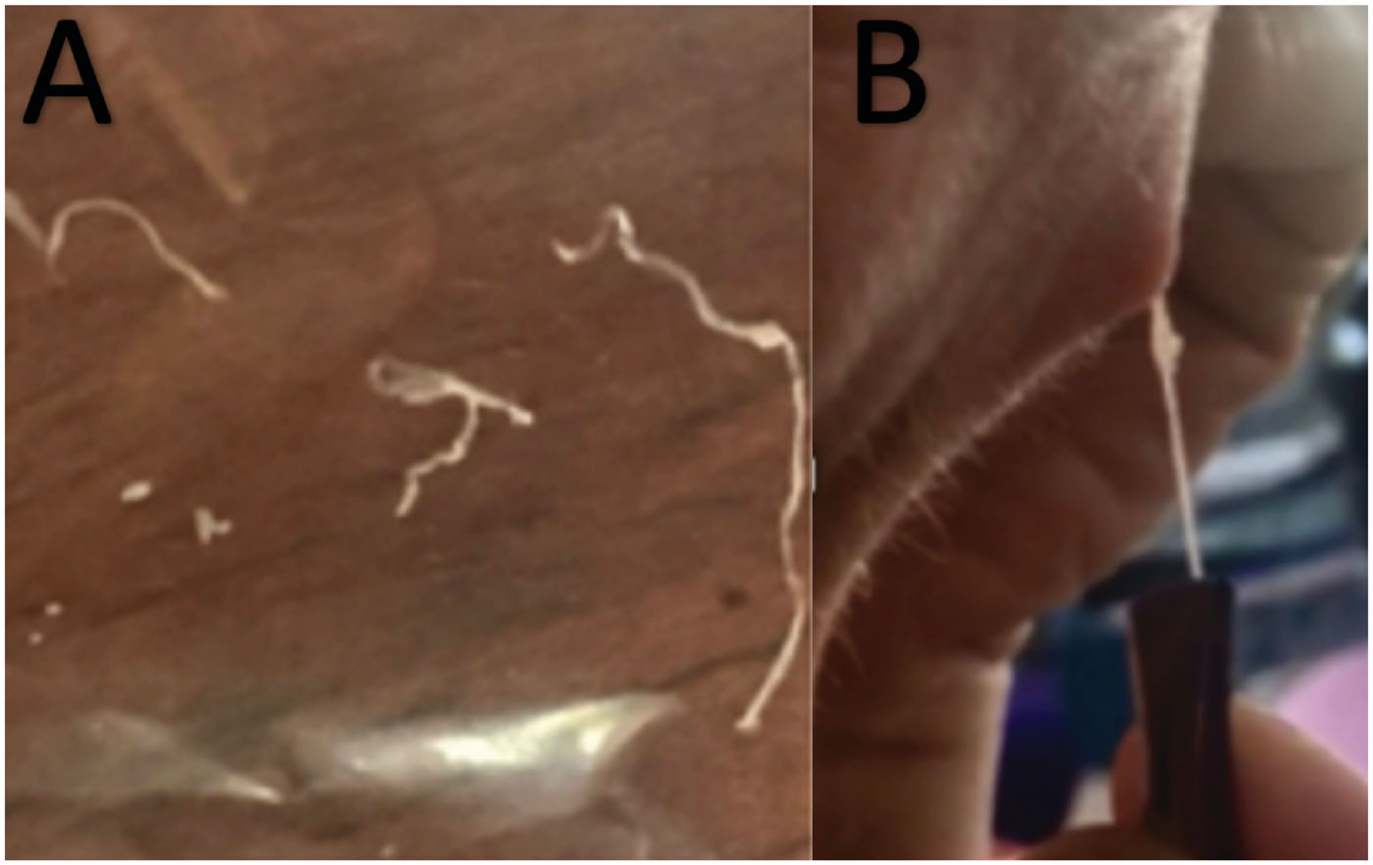
Images (A, B) showing fragments of the *Dirofilaria* nematode being removed from the erupted nodule.

**Figure 5.**
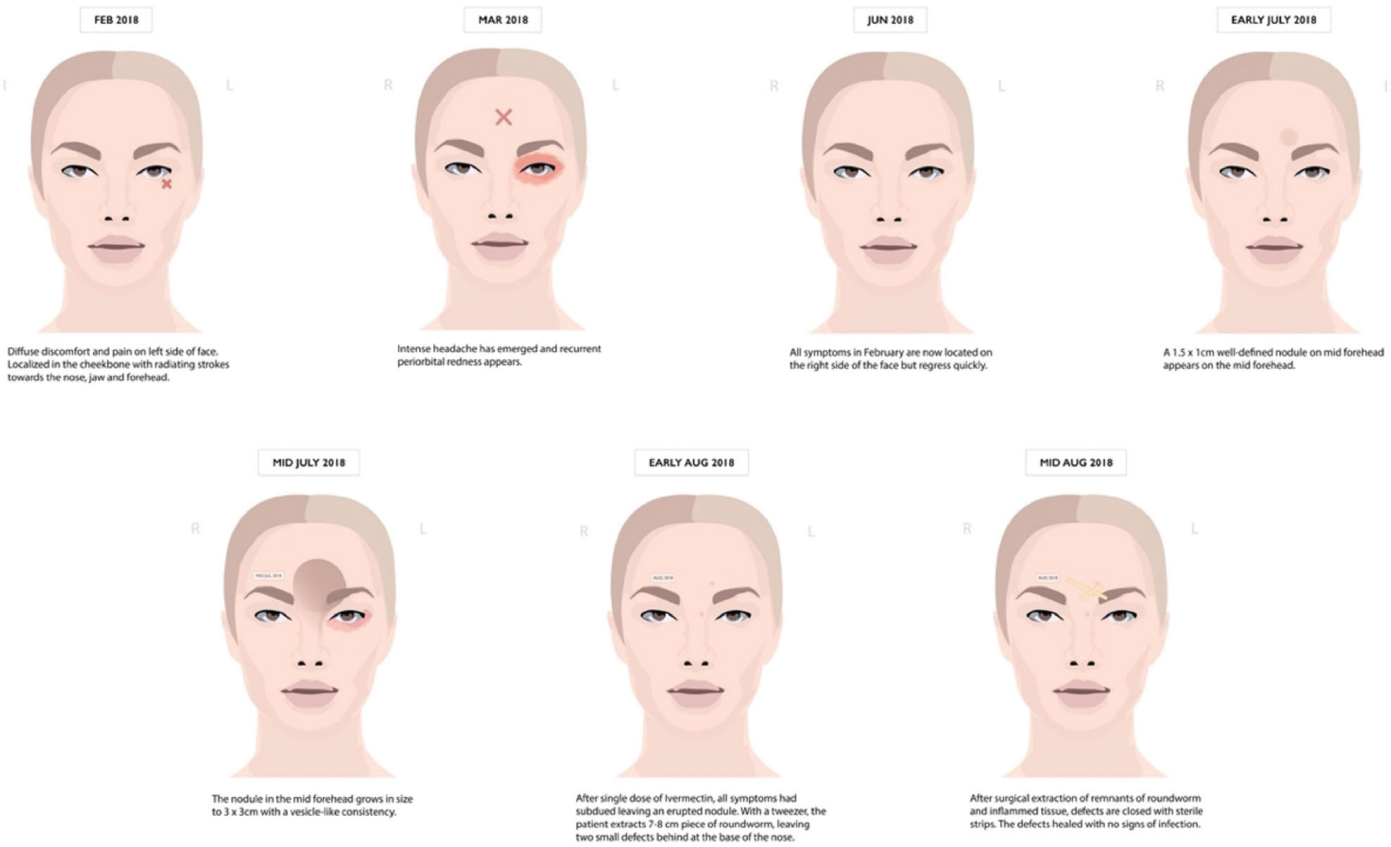
Schematic illustration of the timeline from onset of symptoms. Michelle Alexandra Mistry^©^.

A 46-year-old woman presented to her clinician in February 2018 with diffuse discomfort and pain on the left side of her face. Her past medical history was unremarkable. Within the past five years, she had lived in Denmark but travelled to Spain, Greece, and Sri Lanka. The pain had started at the cheekbone, radiating intermittently out towards the nose, jaw, and forehead. A week later, the pain and discomfort regressed, leaving a small nodule above the left eyelid, with intermittent redness (Figure 1). The clinician suggested idiopathic recurrent periorbital redness. No further work-up was performed, and the condition was left untreated. By mid-March, intense headaches emerged, and the patient was referred to the Emergency Department for evaluation. Physical examination and laboratory results (complete blood cell count, basic metabolic panel, lipid panel, liver panel, blood cultures and infection panel) were unremarkable, and the patient was discharged. No treatment was initiated. By early June, the occasional swelling, redness, and pain reappeared, this time on the right side of the face. Symptoms resolved spontaneously one week later. Five months later, a small 1.5 × 1 cm well-defined, firm nodule developed in the mid-forehead (Figure 2). A course of oral antibiotics (penicillin V 1.5 mill. IU equiv. 660 mg three times per day) was administered on suspicion of an abscess. There was no clinical improvement. Three weeks later, the nodule had increased in size to approximately 3 × 3 cm and had a vesicle-like texture. The patient was then referred to an otolaryngologist, but rhino-laryngoscopy was unremarkable. An infected atheroma was suggested, and a new course of oral antibiotics (clarithromycin 500 mg once daily) was administered. The nodule now had the appearance of a pimple. The pain, redness and swelling migrated to the medial and lower aspect of the left eye. High-resolution ultrasound imaging was then performed which revealed a hypoechoic clarification with a nematode-like structure (Figure 3). The patient was then referred to the Department of Infectious Diseases for further evaluation. Dirofilariasis was suspected based on the findings of additional ultrasound imaging coupled with an extensive medical and travel history and the report of migrating swellings. Ivermectin 12 mg was administered as a single dose. One week later, all symptoms had subdued, leaving a small, erupted nodule. At home, the patient removed nematode fragments measuring approximately 7–8 cm in total (Figure 4(A,B)). Seven months after the initial complaints, remnants of inflamed deeper tissues around the eye were removed by plastic surgeons. On examination, the face was homogenous in colour, texture, and temperature. Two pinpoint defects were observed in the frontal area. One defect was erupted and slightly indurated and located medial to the left supraciliary arch by the eye. The other defect was located as the base of the nose. Under local anaesthesia, an 8-mm incision was made above the tip of the erupted defect, and remnants were sent for microbiological examination (Figure 5). The patient made a full recovery. DNA was extracted using the DNAeasy Blood & Tissue Kit Qiagen, Hilden, Germany), and PCR used the primers FIL 18S (5′-CAAAGTCGTAACAAGGTTTCCG-3′) and FIL_5.8S_R (5′-GCGTCTGCAATTCGCACT-3′) targeting the internal transcribed space 1 region and 5.8 ribosomal RNA gene. Sanger sequencing using the PCR primers as sequencing primers produced a 485 bp-long DNA sequence that was identical to *Dirofilaria* sp. ‘hongkongensis’ strain [16,17] (JX290194 in the NCBI Database); however, sequence coverage was only 48%. Only one reference in the NCBI Database provided 100% coverage, namely KM374817 (*Dirofilaria* sp. ‘Thailand’ clone 7D2), which exhibited 96.52% similarity. Three reference sequences of *D. repens* provided 98% coverage, but only 94.58–94.82% similarity (AB973229, MH059516, and MW349621). We deposited the DNA sequence generated in the present study in the NCBI Database under the accession number (MZ736411).

## Discussion

We report the case of a 46-year-old woman with an unremarkable medical history who initially presented to her primary clinician with pain in her face and diffuse discomfort. After months of delayed diagnosis, the causative agent was identified as *Dirofilaria*.

Patients with subcutaneous dirofilariasis typically report a firm nodule, the classical location being the upper parts of the body and, particularly, around the eye region, followed by the subconjunctival and periorbital areas [12,16]. In our case, the patient presented with initial facial pain and discomfort; then a small nodule near the eyelid appeared, leading to the development of a firm nodule five months later in the middle of the forehead. Bearing similarities to atheromas, the initial symptoms of dirofilariasis are often missed, and hence appropriate treatment is delayed. For our patient, there was a delay of 5 months before the initiation of appropriate treatment, highlighting the need for clinicians to recognize this emerging vector-borne parasitic disease. Within the last five years, our patient had travelled to Spain, Greece, and Sri Lanka, which are all highly endemic areas [12,13,17,18,19]. A limitation of this case report is the lack of detailed morphological description of the nematode. Using PCR and sequencing, the nematode was confirmed to exhibit 100% identity to *Dirofilaria* sp. ‘hongkongensis’, although the two sequences overlapped only partially. *Dirofilaria* sp. ‘hongkongensis’ belongs to the *D. repens* species complex, and it remains unclear whether *Dirofilaria* sp. ‘hongkongensis’ reflects a separate species [20]. Due to the fact that the patient had extracted the worm and discarded it at home, the opportunity for histologic examination was missed. The patient reported that the extracted specimen had a length of 7–8 cm, which could be compatible with a female *D. repens* [14]. Scanning electron microscopy can reveal typical morphological characteristics of the nematode [13,21]. Knott’s test is the golden standard for identifying microfilariae in the blood, though not useful in humans, as microfilariae rarely develop in humans [14]. Eosinophilia may be seen [4,22,23]. Given the nematode’s motility, high-resolution ultrasound imaging is beneficial in identifying the motility prior to morphological identification. Human dirofilariasis is treated by surgical removal, by which the diagnosis can be confirmed [14]. In the scenario of migratory lesions, anthelmintics may prevent the nematode’s migration and promote the formation of a fixed nodule that can be removed by surgery [14]. Given the frequent involvement of the face, surgical intervention is preferred by plastic surgeons to achieve the best possible aesthetic outcome. Genetic characterization of the extracted nematodes is encouraged to provide data for comparison of findings in humans and animals, and evidence for travel-related infections vs. local transmission.

## Conclusion

Dirofilariasis should be suspected also in non-endemic areas if a patient presents with a migrating subcutaneous nodule. Early diagnosis of dirofilariasis is beneficial for the patient, as the condition causes distress and discomfort. The overall health economic and public health impact from improper use of antibiotics is another additional factor to be considered. Plastic surgery is preferred to ensure the best aesthetic outcomes, as the face is often involved.

## Consent

The patient has given written informed consent for the inclusion of material pertaining to them.
